# Isolation, molecular characterization, and genetic diversity of recently isolated foot-and-mouth disease virus serotype A in Egypt

**DOI:** 10.1371/journal.pone.0295319

**Published:** 2023-12-05

**Authors:** Ramy E. El-Ansary, Samy Kasem, Mohamed A. M. El-Tabakh, Yassien Badr, Ahmed S. Abdel-Moneim

**Affiliations:** 1 Faculty of Science, Zoology and Entomology Department, Al-Azhar University, Cairo, Egypt; 2 Department of Virology, Faculty of Veterinary Medicine, Kafrelsheikh University, El Geish Street, Kafrelsheikh, Egypt; 3 Faculty of Veterinary Medicine, Department of Animal Medicine (Infectious Diseases Division), Damanhour University, Damanhour, El-Beheira, Egypt; 4 Department of Microbiology, College of Medicine, Taif University, Taif, Saudi Arabia; Sudan University of Science and Technology, College of Veterinary Medicine, SUDAN

## Abstract

Foot-and-mouth Disease (FMD) is a highly contagious viral disease affecting all hoof-cloven animals. Serotypes A, O and SAT 2 of the foot-and-mouth disease virus (FMDV) are circulating in Egypt. The present study aimed to identify and molecularly characterize the FMDV strains circulating in Northern Egypt during an epidemic that struck the nation in 2022. RNA was extracted from the epithelial specimens, vesicular fluid from affected cattle. The samples were screened using real-time reverse-transcription polymerase chain reaction (RT-PCR) targeting the RNA-dependent RNA polymerase (RdRp) gene. Positive samples underwent individual serotype-specific amplification using primers designed for VP1 of O, A, and SAT 2 serotypes. Subsequently, direct sequencing was performed on the positive samples. The real-time RT-PCR detected positive samples from epithelial and vesicular fluid samples, but not in the blood of infected animals. Out of the 16 samples, seven tested positive for FMDV serotype A. Of these seven positive samples, six were categorized as serotype A-African topotype-G-IV, and these positive samples were isolated in BHK-21 cells, yielding an overt cytopathic effect caused by the virus. In conclusion, it is necessary to sustain continuous surveillance of the evolution of circulating FMDV strains to facilitate the assessment and aid in the selection of vaccine strains for the effective control of FMDV in Egypt.

## Introduction

FMD is a highly contagious transboundary viral disease that affects many cloven-hoofed animals. It results in significant economic losses due to reduced milk production, decreased meat output, the mortality of young animals, medical expenses, and restrictions on the transportation of animals from endemic areas [1–3]. Common symptoms include fever, lethargy, loss of appetite, profuse, often frothy, salivation, and the development of vesicles or blisters on the tongue. While some infected animals may be asymptomatic, they can still carry the virus and transmit it to others. The disease is generally not fatal in mature cattle; however, it increases the risk of abortion in pregnant animals [4].

FMDV is a member of the genus *Aphthovirus*, *subfamily Caphthovirinae*, of the family *Picornaviridae* within the order *Picornavirales*. FMDV is a non-enveloped, single-stranded, positive-sense, non-segmented RNA virus encased in an icosahedral capsid containing around 60 copies of four structural viral proteins (VP1, VP2, VP3, and VP4) [5]. FMDV is divided into seven immunologically distinct serotypes: A, O, C, SAT 1, SAT 2, SAT 3, and Asia 1 [6–8]. Among such serotypes, more than 65 topotypes, genetic lineages, and strains have been identified [5,9]. Africa, Europe-South America (Euro-SA), and Asia are the three recognized topotypes of Serotype A [10,11]. Nucleotide sequences and genetic studies contribute to more than 15% of the genetic variance in viral protein 1 (VP1).

VP1 is responsible for antigenic heterogeneity, serotype specificity, and viral attachment to host cells. The nucleotide and amino acid sequences of VP1 are quite diverse [12]. Because the VP1 coding sequence has been widely employed in molecular epidemiology studies [13], VP1-based diagnostic techniques are widely used [14].

The epidemiology of FMD in Egypt is complicated due to the expansion of local FMD viruses as well as incursions of exotic viral strains from Sub-Saharan Africa and the Middle East [15]. Egypt has been categorized as an FMDV-endemic African nation with many outbreaks since 1958 [16,17]. In Egypt, FMDV serotypes O, A, and SAT2 viruses coexist. According to the World Reference Laboratory for Foot and Mouth Disease (WRLFMD, Pirbright, UK), multiple serotype A topotypes have been reported nationwide. In 2006, the Agriculture Ministry in Egypt notified international public health authorities by reporting to the World Organization for Animal Health of 6 outbreaks of FMDV caused by serotype A in Ismailia and 12 additional outbreaks in 7 other Egyptian governorates; Alexandria, Menofia, Dakahlia, Behera, Dumyat, Fayum and Cairo, during these period the Egyptian and East African strains were comparable [18]. An Asian topotype that fits the Iranian strain (A-Iran-05BAR-08) was originally introduced into Egypt in 2010, and the most recent laboratory report was in 2015 [19]. FMDV serotype A topotype Africa G-IV strain, on the other hand, was discovered in 2012 and has since been reported on several occasions in 2016 and 2018 [19], as well as its dominance in early 2020 [20].

Clinical symptoms are often used to make an initial diagnosis of the condition. Due to natural or vaccine-induced immunity, incomplete symptoms are often ignored in endemic areas [21].

Despite control efforts, various FMDV serotypes and topotypes have been found in Egypt due to live animal and animal product imports and transit, uncontrolled animal movement from neighboring countries, and a lack of quarantine facilities [22]. These factors underscore the importance of continuous monitoring of FMD viral genetic alterations to update FMD vaccine seed strains with currently circulating strains, as well as the need for rapid, reliable, sensitive, and effective diagnostic tests to contain the disease [17].

Real-time RT-PCR (RT-qPCR) is a rapid and highly sensitive molecular diagnostic method for detecting and quantifying nucleic acid targets. Due to its high sensitivity and specificity, it is a serves as a valuable diagnostic tool for FMDV identification [23]. However, achieving significant sensitivity and specificity requires a high degree of complementarity between primers and probes. In the case of FMDV, which exhibits considerable sequence variability, mismatches between primer and probe sequences can result in false-negative results or amplification failure. Therefore, it is essential to regularly review primer and probe sequences to ensure their suitability for their intended purpose [24].

Periodic FMDV mutations lead to significant antigenic changes and the emergence of new topotypes and lineages, potentially resulting immunization failure [25]. Consequently, the development of molecular-based technologies for the early and rapid detection and differentiation of circulating FMDV serotypes is crucial during persistent endemic epidemics [26]. In the current study we aim to genotype FMDV strains from the recent 2022 outbreak in different districts in northern Egypt.

## Materials and methods

### Animals

Naturally infected cattle show clinical symptoms suggestive of FMD, including salivation, the appearance of vesicles on the tongue and in the mouth, depression, and loss of appetite. Representative samples from examined dairy farms were collected from Behera (n = 8), Kafr El-Sheikh (n = 4), and Gharbiya (n = 4) during the period between July and October 2022. Dairy farms in Behera and Gharbiya were vaccinated with a local polyvalent inactivated vaccine developed by the Veterinary Serum and Vaccine Research Institute (VSVRI) in Cairo, Egypt. In contrast, the vaccine status of animals in Kafr El-Sheikh governorate was unknown.

### Samples

The samples were collected and processed in accordance with the World Organization for Animal Health (OIE) standard criteria [27,28]. Briefly, epithelial tissue was collected from an unruptured or recently ruptured vesicle on the tongue or the buccal mucosa and placed in virus transport medium containing penicillin (1000 IU), neomycin sulfate (100 IU), polymyxin sulfate (50 IU), and myostatin (100 IU). Vesicular fluid was aspirated from unruptured vesicles using a sterile needle and transferred to a sterile sample container. All samples were kept in an icebox to maintain the cold chain temperature during transport to the laboratory. Sterile sand was used to crush the tissue samples. A 10-minute centrifugation at 3000 rpm for 10 minutes at 4°C clarified the tissue homogenate. The supernatant was filtered using syringe filters (0.2 μm filter, Corning) and then incubated for three hours at room temperature with a 1% antibiotic solution (100 IU/mL Penicillin and 100 mg/mL Streptomycin). The processed sample solution was then stored at -80°C until virus isolation and molecular analysis.

### Virus isolation

In constrained biosafety level-2 environments, the produced materials were injected into the virus-adapted kidney cell line of Baby Hamster Kidney (i.e., BHK-21) and were used for FMDV isolation [29]. Confluent monolayer cell cultures were inoculated with the produced materials, while control cells consisted of non-infected cells. The inoculated cells were cultured in 25 cm^3 cell culture flasks containing Earl’s salts, 10% fetal calf serum, 100 IU/ml penicillin, and 100 mg/ml streptomycin. The growth media supernatant in the flasks was decanted, and the cell monolayer was washed three times with sterile phosphate-buffered saline before gently applying 0.5 ml of the viral sample to the cell monolayer. The flasks were incubated at 37°C for 90 minutes in a 5% CO2-supplied incubator, with gentle tilting every 10 minutes. The flasks were monitored for viral cytopathic effects on the BHK-21 cell monolayer for 24 to 72 hours. When cytopathic effects (CPE) were observed, infected cells were harvested, subjected to three freeze-thaw cycles, and then passaged three times consecutively.

### RNA extraction

Viral RNA was extracted using the EasyPure® viral RNA kit (TransGen Biotech, Beijing, China, Cat. No. ER101-01) from a 200 μL processed sample, following the manufacturer’s instructions. Viral RNA was eluted in 30 μL of RNase-free water and stored at -80°C.

### FMD screening using real-time RT-PCR (qRT-PCR)

For the detection of FMDV, primers and probes directed toward the conserved 3D gene (conserved catalytic core domain of RNA-dependent RNA polymerase (RdRp)), were used as previously described [29] (Table 1). TransScript® Probe One-Step qRT-PCR SuperMix (TransGen Biotech, Beijing, China, Cat. No. AQ221) was employed for 3D gene amplification, following the manufacturer’s instructions. The cycles consisted of reverse transcription at 45°C for 5 minutes, followed by initial denaturation at 94°C for 2 minutes, with forty cycles of 94°C for 30 seconds and 60°C for 30 seconds. Samples with a cycle threshold (Ct) of fewer than thirty were used for standard one-step RT-PCR.

**Table 1:** Primers used in real-time RT-PCR of 3D gene, one-step RT-PCR and sequencing reaction of 1D gene of FMDV.

Serotype	Primer designation	Primer sequence (5’-3’)	Amplicon size	References
All serotypes (3D gene)	3D-F	ACTGGGTTTTACAAACCTGTGA	106 bp	Callahan et al., 2002
	3D-R	GCGAGTCCTGCCACGGA		
	3D-Probe	TCCTTTGCACGCCGTGGGAC		
O	O-Egy-F	CCTCCTTCAAYTACGGT	283 bp	El-mayet et al., 2020
A	A-Egy-F	GGAATCWGCAGACCCTGTC	750 bp	
SAT2	SAT2-Egy-F	TGAYCGCAGTACACAYGTYC	666 bp	
A,O, and SAT2	NK61–2BR	GACATGTCCTCCTGCATCTG	-	

### One-Step Reverse-Transcription Polymerase Chain Reaction (RT-PCR)

The TransScript® One-Step RT-PCR SuperMix amplified the VP1 gene using three selective primers for the three serotypes (A, O, and SAT2) (TransGen Biotech, Beijing, China, Cat. No. AT411) as previously described [30] (Table 1). In summary, a 25 μL PCR reaction mixture consisting of 5 μL of the RNA template, 12.5 μL of the One-Step Reaction Mix, 0.4 μL of the One-Step Enzyme Mix, and 3.1 μL of RNase-free water was combined with 2 μL of each primer (10 pMol) as instructed by the kit manual. The target genes were amplified using a BIO-RAD® PCR system T100 thermocycler (BioRad, Hercules, California, USA). The following conditions were employed for the one-step RT-PCR: 45°C for 25 min (reverse transcription step), 94°C for 5 min (denaturation), followed by 40 repeated cycles of 94°C for 45 sec (denaturation) and 60°C for 1 min (annealing), except for the SAT2-specific detection primer at 50°C for 45 sec (annealing) and 72°C for 45 sec (extension), followed by a final extension at 72°C for 10 min and scheduled for a final hold at 4°C. The PCR results were then subjected to 1.5 percent agarose gel electrophoresis using Tris Borate EDTA buffer (1X) stained with ethidium bromide. The RT-PCR products and DNA ladder were loaded into the appropriate wells, and the agarose gel was examined and analyzed for FMDV-specific bands using the Molecular Imager Gel DocTM XR + Imaging system (BIO-RAD) and Image labTM gel image analysis software. The amplified RT-PCR products were purified from the gel using the QIAquick Gel Extraction Kit (Qiagen, USA, Cat. No. 28704), following the manufacturer’s instructions. The QubitTM dsDNA HS test kit (Molecular Probes, Life Technologies, USA) was used in combination with the Qubit® 2.0 fluorometer for precise DNA quantification.

### DNA sequencing

The purified PCR products were sequenced using a dideoxy chain termination approach with the same primers. The sequencing reaction was prepared using the BigDye® Terminator v3.1 Cycle Sequencing Kit (Thermo Fisher, USA). For sequencing, the thermal cycling conditions included 96°C for 1 minute, followed by 25 cycles at 96°C for 10 seconds. The T-100TM Thermal Cycler was set to 5 seconds at 50°C and 2 minutes at 60°C (Bio-Rad, USA). The sequencing reaction product was purified using Centri-SepTM Spin Columns (Thermo Fisher, USA), which were then electrokinetically injected into capillary electrophoresis systems 3500 Genetic analyzers (Applied Biosystems, USA).

### Phylogenetic analysis

We utilized NCBI’s Basic Local Alignment Search Tool (BLAST) to compare nucleic acid and amino acid sequences. Multisequence alignment was performed using MEGA 5.1. For phylogenetic analysis, we employed the Maximum Likelihood method with 1000 bootstrap replications and applied the Tamura-Nei model [31,32].

## Results

### Clinical signs

During the outbreak investigation and sample collection, the study population observed salivation and vesicle formations in the oral cavity and interdigital spaces. FMD lesions on the mouth included sores, destruction of the upper and lower pad regions, and tongue lacerations, while foot abrasions included wearing away in the interdigital space. Study groups lagged behind healthy cattle in terms of nutrition and milk output.

### Virus isolation

**Fig 1:**
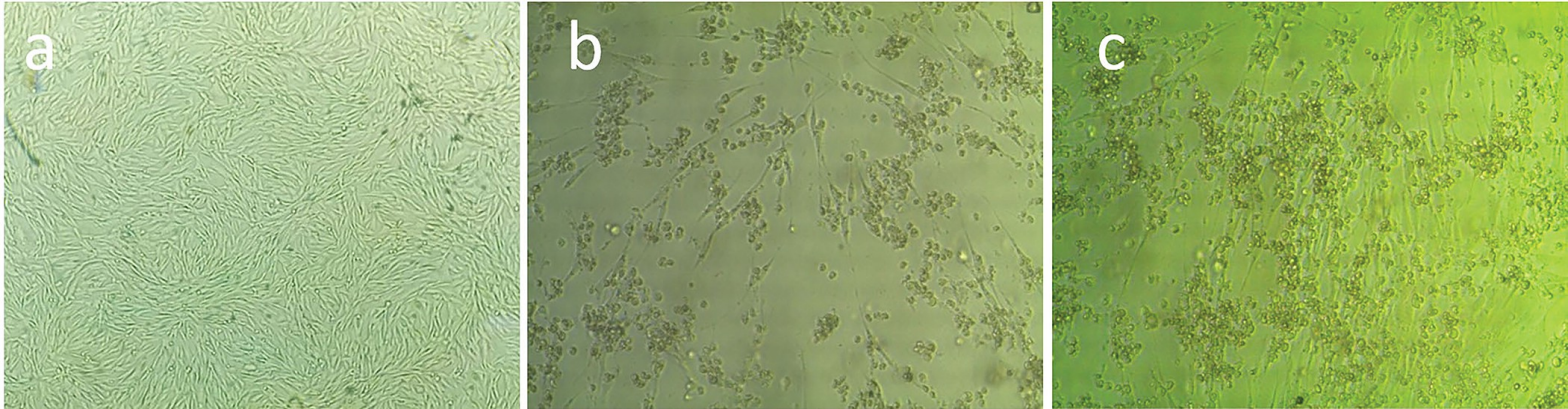
Isolation of FMDV on BHK-21 cell line. (a) Control BHK-21 cell line (cell without FMDV infection), (b & c) positive BHK-21 cell line induced CPE inoculated with FMDV outbreak samples with Ct values ranged from 17.21 to 23.38 showing rounding, granulation or swelling.

The virus was cultured from seven FMDV-positive samples (six epithelial and one vesicular) selected based on their Ct values. Cytopathogenic effects (CPE) of FMDV on BHK-21 cells were observed as cell rounding, swelling, granulation, and monolayer detachment from the cell culture flask surface. Cells were severely damaged and eventually died within 24–72 hours following injection, indicating the presence of an infectious virus. However, samples with no CPE did not cause any morphologic alterations in BHK-21 cells. Positive viral isolation findings on BHK-21 cells were confirmed using related samples. Ct values varied from 17.21 to 23.38 (Fig 1). However, the remaining FMDV clinical samples (n = 9) did not exhibit any CPE on BHK-21 cells, with Ct values exceeding 29.75.

### FMDV screening using qRT-PCR

Using qRT-PCR, the 16 collected samples were screened for FMDV, and seven (43.7 percent) were found to be positive when pan-FMDV 3D primers were used. Positive samples had Ct values ranging from 17.21 to 23.38 (Table 2).

**Table 2:** Real time reverse transcriptase polymerase chain reaction on clinical samples from affected cattle that showed clinical signs relevant to FMDV infection.

Date of collection	Location	Sample ID	Cycle Threshold (CT)^c^	Molecular Tests results (Blood)	Molecular Tests results (tissues)	Accession number ^e^
16/07/2022	Behera	C1	33.87	Negative	Negative	**-**
		C2	19.62	Negative	Positive	**OQ316637**
		C3	31.19	Negative	Negative	**-**
23/07/2022	Behera	C4	17.73	Negative	Positive	**OQ316638**
		C5	33.23	Negative	Negative	**-**
		C6	29.75	Negative	Negative	**-**
		C7	30.14	Negative	Negative	**-**
28/07/2022	Gharbiya	C8	32.35	Negative	Negative	**-**
		C9	33.45	Negative	Negative	**-**
		C10^a^	17.45	Negative	Positive	**OQ316639**
12/08/2022	Gharbiya	C11	19.59	Negative	Positive	**OQ316633**
16/08/2022	Kafr El-Sheikh	C12 ^b^	31.12	Negative	Negative	**-**
		C13 ^b^	17.21	Negative	Positive	**OQ316634**
21/08/2022	Kafr El-Sheikh	C14	17.59	Negative	Positive	**OQ316635**
11/09/2022	Kafr El-Sheikh	C15	18.22	Negative	Positive	**OQ316636**
14/09/2022	Behera	C16^d^	31.76	Negative	Negative	**-**

Samples: Both skin scape from the affected lesion and heparin treated blood were collected from infected animals.

^a^The sample was vesicular fluid and heparin treated blood.

^b^Animals were vaccinated by locally prepared vaccine except for two farms in Kafr El-Sheikh with unknown vaccination status (^b^).

^d^ Pregnant animal.

^e^ Accession numbers of the VP1 gene of the samples showed positive in real RT-PCR and positive for serotype specific one-step RT-PCR.

### Sequencing and genetic characterization

**Fig 2:**
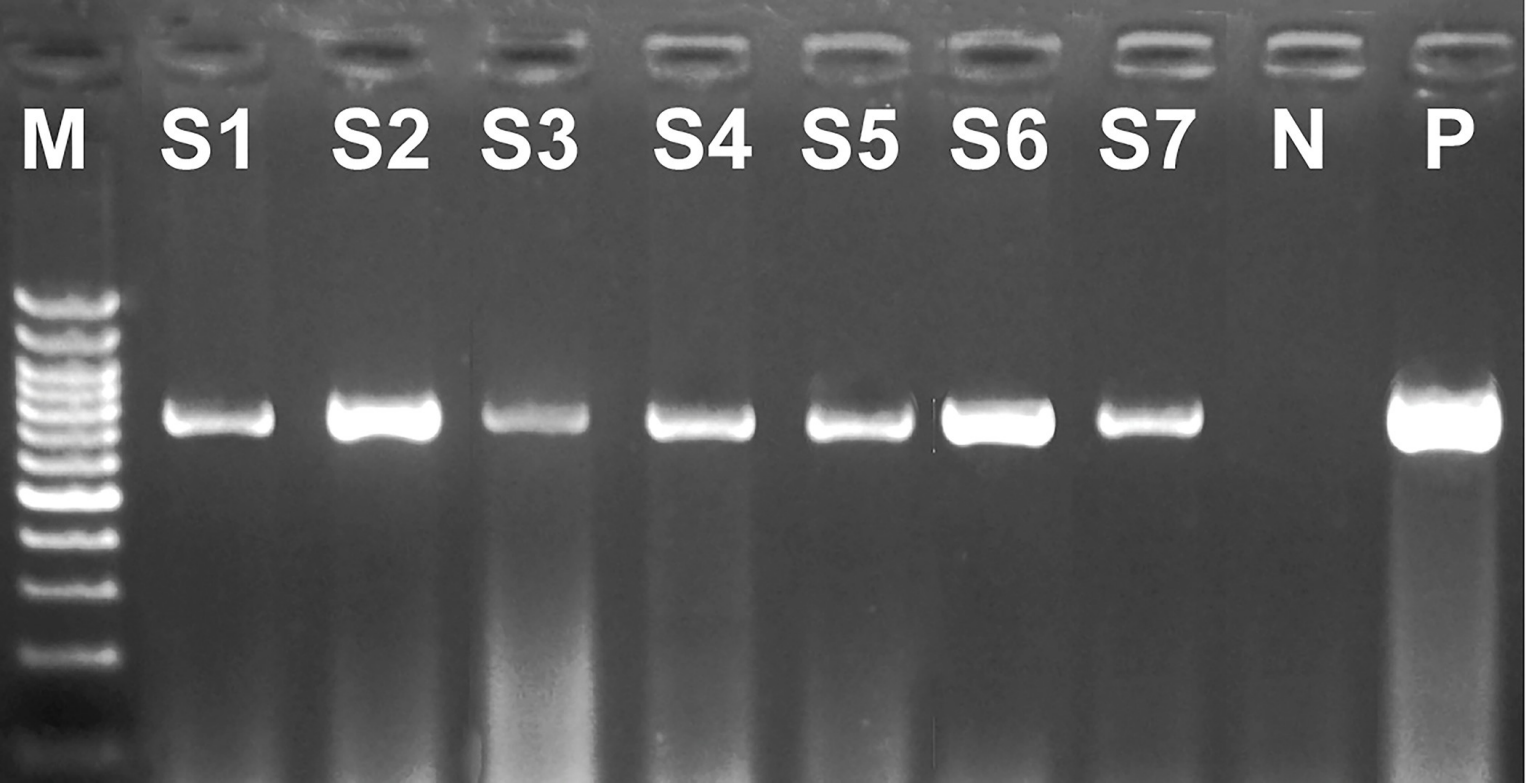
Gel electrophoresis of RT-PCR amplicons of a local FMDV strain from clinically diseased cattle using serotype A specific primers. Lane M: High molecular weight nucleic acid marker (100 bp); lanes S1-S7, tested samples which are epithelium tissues and vesicular fluid samples collected from different governorates in 2022 from July to October; Lane 8, (N) negative control and Lane 9, (P) positive control for FMDV.

FMDV-positive qRT-PCR specimens were further investigated using serotypes A, O, and SAT2 specific primers. In cases where samples could not be identified using the previously published serotype-specific primers, all seven pan-FMDV positive isolates were found to be serotype A positive, as confirmed by conventional RT-PCR (Fig 2 and S1 Raw image).

The partial-length of 1D (VP1 protein) gene sequences of 6 out of 7 FMDV isolates obtained in this study was successful (accession numbers OQ316633-OQ316638). BLAST analysis for VP1 encoding of the recently detected and currently circulating Egyptian FMDV.

**Fig 3:**
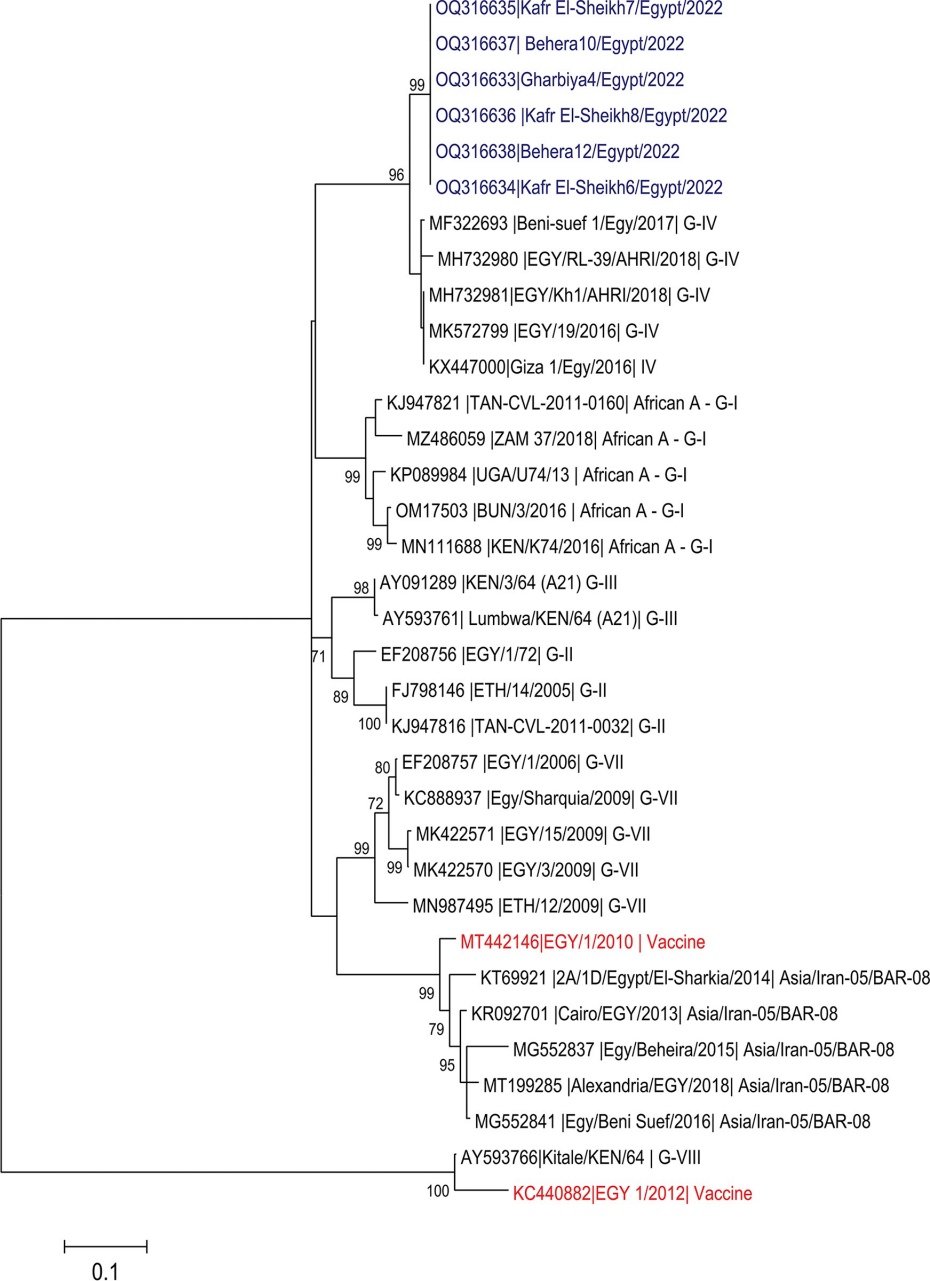
Phylogenetic tree of the VP1 nucleotide sequences in comparison to other FMDV serotype A sequences. Red sequences are related to the vaccines, blue sequences are those identified in the current study. Phylogenetic tree was created using MEGA 5.1 with Maximum Likelihood method with 1000 bootstraps replications.

The strains in our study exhibit a close relationship with sequences of Egyptian FMD viruses of serotype A, subclade IV, isolated between late 2015 and 2022. They were closely related to Egyptian A serotypes (IV subtype) detected in different parts of northern and southern Egypt. Another serotype A FMDV circulating in Egypt includes Asia/Iran-05/BAR-08, GI, G-II, G-III, G-VII and G-VIII. Two strains of FMDV serotype A are in use as seed virus strains of local vaccines, including MT442146|EGY/1/2010, which belongs to the Asia/Iran-05/BAR-08 serotype, and KC440882|EGY 1/2012, which is found to be related to (AY593766|Kitale/KEN/64 | G-VIII) (Fig 3).

**Fig 4:**
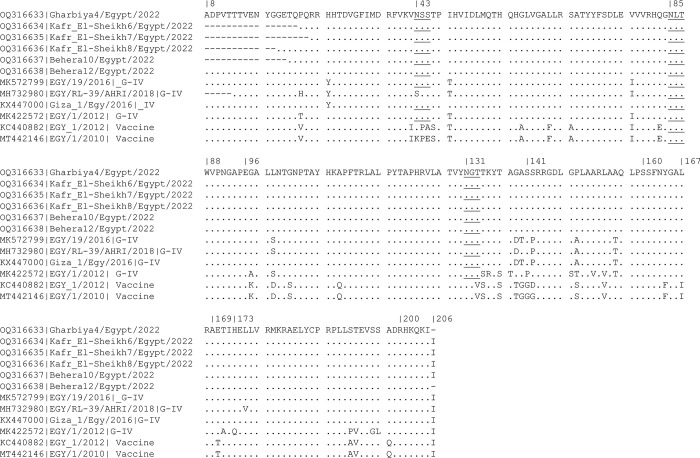
Deduced amino acid sequences of the VP1 protein of the strains detected in the current study in comparison to selected FMDV strains and serotype A vaccine sequences. Dots mean identical sequences. Underlined sequence denotes to N-glycosylation sites. 141–160 is the G-H loop, and 200–211 is the C-terminus area.

The strains identified in the current study showed three N-glycosylation sites at positions 43, 85 and 131. All strains in the current study were identical to each other but showed amino acid substitutions to other G-IV subtypes. They exhibited 31 and 32 amino acid substitutions from KC440882|EGY_1/2012 and MT442146|EGY/1/2010, respectively, which also lack the third N glycosylation site present in the current (Fig 3). P149 is present in all strains in the current study (Fig 3). P149S was detected in the two vaccine strains, while P149 to A (three Egyptian strains) and P149T (in a single Egyptian strain) (Fig 4).

## Discussion

FMDV genome displays a high mutation rate and evolves rapidly (2, 3), leading to the subsequent emergence of numerous subtypes [33]. Changes in the VP1 hypervariable region result in the evolution of numerous antigenic variants. Serotype A comprises three major subtypes: Africa, Asia, and Euro-SA. The Africa subtype encompasses GI to G-VIII, while the Asia subtype consists of 10 clades: (A15, A22, G-VII, Iran-5, Iran-87, Iran 96, Iran 99, Oak-09, Sea-97, and Thai-87). The Iran-5 clade contains 17 subclades. The Euro-SA subtype is comprised of four clades: A-81, A12, A24, and A5 [11]. The emergence of FMDV variants is accelerated by immune pressure or immune selection during infection of partially immunized animals [33–35].

In Egypt, repeated epidemics of FMD persist despite the intensive use of polyvalent FMDV vaccines, including locally isolated vaccine strains containing serotypes O pan Asian II (EGY/2010), An Iran 05 (A/EGY/1/2012), A/ KC440882|EGY 1/2012, SAT2 (EGY/Gharbia/2012), and SAT2 (LIB/2018). This suggests there may be a vaccination failure. Possible causes of vaccination failure include the presence of immunosuppressive disorders that hinder the animal’s ability to develop a satisfactory immune response, improper storage and transport of the vaccine, and immune evasion due to the presence of different vaccine variants as previously reviewed [36]. This fact means that FMDV continues to be an endemic disease in the country. Immunization efforts have been effective in reducing FMD incidence; however, FMDV strains continue to circulate due to unregulated animal movement. Furthermore, Egypt’s unique geographical location increases the likelihood of the introduction of circulating strains from Asian and East African nations into the country. In the past decade, at least eight FMDV serotype strains have been identified (A, O, and SAT2), including (A-Iran 05, A-African genotype IV, A-African genotype VII, O-Pan Asia II, O East Africa, SAT2 Ghb-12, SAT2 Lib-12, and SAT2 Alx-12) with alternating displacement and an annual winter peak of two to three circulating strains and a dominant circulating strain [30]. According to the first 12 hits of BLAST data from this lineage, all sequenced FMDV isolates in this study were genetically classified as serotype A of genotype IV of the African topotype [37].

Antigenic properties of FMDV strains have been found to be associated with specific amino acid motifs in the VP1 protein: 43–45, 83, 96, 141–160 (G-H loop), 169–173, and 200–211 (C-terminus), as previously determined in serotype A [38–42]. In the current study, current vaccine strains were found to have different amino acid substitutions from the strain identified in the current study, including T133V, T137S, G139T, A140G, N43K, Q83E, S141G, S142D/G, P149S, L154V, and E170T. This highlights the possibility that the current vaccine strains may offer reduced protection against the recent circulating strains. The substitution from P149 to S149 affects the antigenicity of the A22 vaccine [41]. However, sequence homology provides an indication of the relatedness of the virus strains circulating in the region and their relation to the vaccine strains, but it serves as only an indirect guide. Therefore, one should primarily rely on cross-neutralization studies to assess the effectiveness of vaccine coverage and cross-neutralization. The latter is more suitable for determining the efficacy and cross-protection of the vaccines in use.

In conclusion, the current study underscores the ongoing challenges in controlling FMDV infection in Egypt, as it highlights the need for continuous monitoring and vaccine strain selection to effectively manage FMDV in the region.

## Supporting information

S1 Raw image Raw gel electrophoresis of RT-PCR amplicons of a local FMDV strain from clinically diseased cattle using serotype A specific primers. (JPG)
