# Supplementary figures and images for: Isolation, molecular characterization, and genetic diversity of recently isolated foot-and-mouth disease virus serotype A in Egypt
Source: PLoS One. 2023 Dec 5;18(12):e0295319. doi: 10.1371/journal.pone.0295319 (PMC10697586; doi:10.1371/journal.pone.0295319)

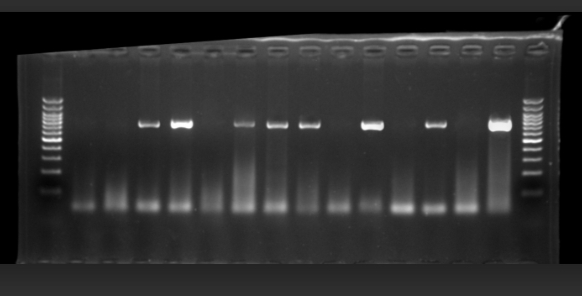

Supplement: S1 Raw image — (JPG) [file pone.0295319.s001.jpg]
